# Latent Syphilis

**Published:** 1864-01

**Authors:** 


### LATENT SYPHILIS.

In reply to an inquiry addressed to medical practitioners in all countries "On the influence which an Anti-Syphilitic Mercurial Treatment of Parents has on the Health of their Children," Hebra draws certain deductions from ten cases occurring in his private practice, among which conclusions we find the following: "Syphilis may remain latent in the body, without discovering itself by any symptoms, and first betray itself by the syphilitic affection of the children."--(*Year-book of Surgery*, 1860, p.322.) By which is meant, that a man may contract syphilis, and fancy himself well, and yet after marriage, at some distant period, while in apparently perfect health, beget children, who may become the subject of the syphilitic taint in a constitutional form. The great difficulty in obtaining perfectly reliable information on these questions from patients must be admitted by all, but still, with care, we may arrive at a close approximation of the truth.

Hebra states a fact which has been recognized by many in the surgical practice at St. Bartholomew's Hospital for a long period. The two following cases, which came under Mr. Coote's care on August 15, 1863, illustrate, he considers, Hebra's views:—

CASE 1.—Jane M., aged 27, a healthy looking young woman, married three years, came to the hospital bringing an infant covered from head to foot with syphilitic psoriasis. She said that the eruption showed itself a few days after birth, and the child had since pined and become emaciated. She added that she had one other child about eighteen months previous, which was born dead. The closest inquiries were made as regarded both her own health and that of her husband. She replied readily and frankly, with a full knowledge of the purpose of the question, and assured us that no illness had shown itself on either herself or her husband in any way during their married life. The child was ordered two grains and a half of mercury and chalk twice a day, and is going on favorably.

CASE 2.—Elizabeth A., aged 16, a slightly made, pallid child, living with her parents, came to the hospital with a circular and perforating phagedænic ulcer of the soft palate, the size of a sixpence, with tawny surface and inflamed circumference. There were adhesions of the left iris around the pupilary margin, but no indications of recent iritis. She was questioned with care, consideration being of course shown to her youth, and it was obvious that she was innocent of any irregularity. It was also denied that either parent had ever suffered from syphilitic disease during married life. The edges of the ulcer were touched with nitric acid diluted with five parts of water. She was ordered three grains of iodide of potassium in sarsaparilla. The case is going on well.

Hebra adds that the reverse may happen, viz., fathers infected with general syphilis may neither infect their wives nor produce syphilitic children. In illustration of this statement I mention the following case:—

CASE 3.—Thomas W., 38, a respectable man, contracted syphilis twelve years ago, and only on that occasion. He had an indurated chancre at the reflection of the prepuce on the glans penis. It was treated with mercury and disappeared, and the man remanied well for two years, when he became the subject of syphilitic lepra, chiefly affecting the arms, chest, and head. In course of time the eruption disappeared, and seven years ago he married. His wife and children are in good health, but he is subject to syphilitic ulceration of the tongue and fauces, and to occasional attacks of lepra in a slight form.—*Med. Times and Gaz., Sept.* 15, 1863.

